# Elevated Levels of Serum Vascular Endothelial Growth Factor-A Are Not Related to NK Cell Parameters in Recurrent IVF Failure

**Published:** 2017

**Authors:** Rhea Bansal, Brian Ford, Shree Bhaskaran, Meenyau Thum, Amolak Bansal

**Affiliations:** 1- Department of Immunology and Allergy, St Helier Hospital, Carshalton, United Kingdom; 2- Fertility Clinic, Lister Hospital, London, United Kingdom

**Keywords:** NK cell cytotoxicity, Recurrent failed IVF, Soluble fms-like tyrosine kinase 1 (s-FLT-1), Vascular endothelial growth factor

## Abstract

**Background:**

Vascular Endothelial Growth Factor and NK cells have an interrelated role in angiogenesis that is critical for placentation and success of *in vitro* fertilization. An attempt was made to assess a possible relationship between the two in this study.

**Methods:**

A case control study was performed comparing the serum levels of VEGF-A and its receptor VEGF-R1 with levels of NK cells, activated NK cells and NK cytotoxicity in 62 women with Repeated Implantation Failure (RIF). The healthy control group consisted of 72 women of similar age, without known issues in achieving pregnancy or evidence of autoimmunity. Levels of VEGF-A and VEGF-R1 were quantified by ELISA methods with standard curve interpolation. NK cell subsets were determined with flow cytometry using fluorescent-tagged anti-CD56, anti-CD16, anti-CD3 and anti-CD69. NK cytotoxicity was performed by incubating peripheral blood mononuclear cells and K562 cultured cells with propidium iodide, steroid, intralipid and intravenous immunoglobulin, using previously described methods. Statistical analysis involved Mann-Whitney-U and Spearman’s rank correlation testing with p-values defined as <0.05.

**Results:**

It was found that VEGF-A levels were significantly raised in women with RIF compared to healthy controls (362.9 *vs*. 171.6 *pg/ml*, p<0.0001), with no difference in VEGF-R1 levels between groups (1499 *vs*. 1202 *pg/ml*, p=0.4082). There was no correlation between VEGF-A or VEGF-R1 and the absolute levels of circulating NK cells, CD69 activated NK cells or NK cytotoxicity.

**Conclusion:**

The absence of correlation between VEGF-A or VEGF-R1 and NK cells suggests VEGF secretion and regulation is independent of NK cell activity in RIF.

## Introduction

Recurrent *in vitro* fertilization failure or Repeated Implantation Failure (RIF) has been defined in the UK as failure of pregnancy after 2–6 IVF cycles, of which 3 of those would be fresh cycles (rather than involving frozen embryos) involving transfer of more than 6 embryos (1). The precise definition remains somewhat vague and has been recently debated (2). Recurrent spontaneous abortion (RSA) is defined as 2 or more miscarriages occurring consecutively in the first or early second trimesters of pregnancy. It can affect up to 1 in 20 women of reproductive age and in half of these cases, the precise cause remains unclear despite intensive investigation. It is now thought that a hypoxic placental environment caused by impaired angiogenesis (3) and related to endothelial dysfunction may play a significant role here (4, 5).

The Vascular Endothelial Growth Factor (VEGF) family are potent angiogenic and vasculogenic growth factors. VEGF has widespread effects not limited to the circulatory system. These include bone formation, haematopoiesis and tissue remodelling. These functions are reflected in the widespread production of VEGF by tumour cells, endothelial cells, macrophages, platelets and keratinocytes (6). In relation to reproduction, VEGF is a key mediator of angiogenesis within the uterus, endometrium and placenta (7). VEGF-A is the primary form of VEGF expressed in the placenta, which is involved in placental angiogenesis as well as the spiral artery remodelling process (8). Both processes are critical for proper nourishment of the developing embryo. The key stimulus for VEGF-A mRNA production is hypoxia, which occurs via hypoxia-inducible factor 1α (9); VEGF-A primarily binds to two receptors, receptor-1, also known as FLT-1 (fms-like tyrosine kinase 1) and receptor-2, also known as KDR (kinase insert domain receptor)- the former was discussed in more detail throughout this article (10). R1/FLT-1 is expressed on monocytes/macrophages as well as placental trophoblast cells. Its expression is upregulated in the presence of hypoxia (11).

Uterine NK cells are of critical importance in normal pregnancy for the processes of spiral artery remodelling and placentation (12, 13). They are also involved in the regulation of inflammatory Th17 cells at the maternal-fetal interface in the placenta (14). However, increased numbers and activation of peripheral NK cells (15–18), endometrial NK cells (19) and reduced numbers of decidual NK cells with KIRs specific for HLA-C (20) have all been associated with recurrent IVF failure and also recurrent miscarriage (RM). The numbers of decidual NK cells with ultrastructural changes and their distribution along uterine blood vessels also appear to be increased in women with RM (21).

Reduced VEGF levels have been considered important in RM (22) and RIF and preeclampsia. Soluble FLT, formed by alternative splicing of the R1/FLT-1 mRNA, may have a similarly relevant role. s-FLT is known to have potent anti-angiogenic properties, with animal models demonstrating its ability to inhibit both VEGF-A and placental growth factor (PlGF) and induce pre-eclampsia (23, 24). Indeed, R1/FLT-1 and sFLT-1 share the same ligand binding domain, but sFLT-1 lacks the membrane-spanning and intracellular domains. Several reports have found circulating levels of sFLT to be raised in women with threatened abortion (25) or RM (26). Detecting circulating complexes of VEGF and sFLT that may affect the analysis of circulating levels of both factors has not been reported. From a genetic perspective, several SNPs that are associated with reduced VEGF functional activity have been variously reported from fertility centers around the world (27). It is presently unclear whether the relevant foetal VEGF SNPs are more important than the maternal ones (28).

In mice, decidual NK cells (*i.e*. at the fetal-maternal interface) have been shown to secrete VEGF and were considered to be derived from precursors recruited to the uterus (29). Additionally, pregnant Ly49 knock-out mice had reduced NK cell infiltration and impaired decidual angiogenesis that was associated with reduced expression of VEGF (30). In humans, however, decidual NK cells have been shown to express VEGF and this was little altered between the first and second trimester (31). In the peripheral blood, NK cells have been recently reported to express VEGF and reduced levels were noted in pregnant women with gestational diabetes (32), a group known to have impaired reproductive outcome.

From the above, it appears that both NK cells and VEGF are important for placentation. Importantly, both decidual and peripheral blood NK cells are capable of secreting VEGF. Therefore, in this study, an attempt was made to examine the total peripheral blood NK cell count, CD69 activated NK cell count and NK cell cytotoxicity with the circulating levels of VEGF-A and VEGF-R1 in women with RIF to see if these may be related.

## Methods

### Study Design and Subject Selection:

A case-control design was used to evaluate the serum levels of VEGF-A, and soluble VEGF-R1 in women with recurrent IVF failure (RIF) and healthy controls. The patient group consisted of sixty two women referred to the Reproductive Immunology Centre for immunological evaluation of infertility or recurrent pregnancy loss. Referral came via the fertility clinic at the Lister Hospital in London. RIF was defined as the absence of pregnancy following 3 cycles of IVF using good quality fresh embryos.

**Figure 1.**
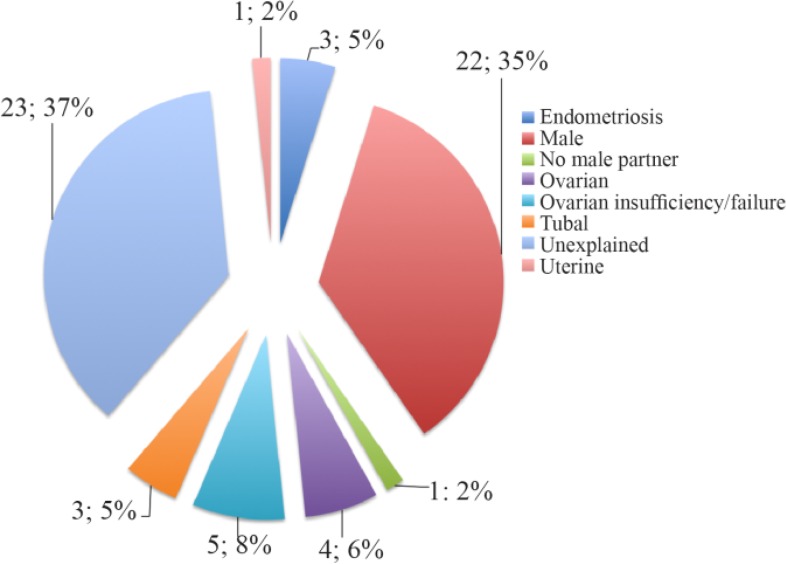
Primary Diagnosis of Infertility in the RIF Group

Table 1 details the demographic and clinical features of the patient group including information on IVF cycles undertaken after sample collection and outcomes. Samples were collected in the first half of the menstrual cycle and at least two months after any hormonal reproductive manipulation. Figure 1 also shows the primary diagnoses attributed to these women, with regard to their subfertility status. Women with positive tests of autoimmunity, endocrine dysfunction or an alternative defined cause for their subfertility were excluded. Thus, all women had no clinical evidence of a systemic connective tissue disorder or anti-phospholipid antibody syndrome, negative anti-nuclear antibodies on Hep2 cells, no anti-cardiolipin or anti-thyroid peroxidase antibodies, and had normal T4 and TSH.

**Table 1.** RIF group demography and outcomes

**Number of patients, n**	62
**Median Age, years (*min*, max)**	39 (29, 45)
**Number of Pregnancies following IVF cycles, n (%)**	39 (41)
**Number of Live Births resulting from pregnancies, n (%)**	24 (61.54)
**Number of Miscarriages resulting from pregnancy, n (%)**	15 (38.46)

The healthy control group comprised seventy two women of comparable reproductive age, without evidence of reproductive difficulty and no evidence of autoimmunity as indicated above for the RIF group. They had normal full blood count and normal ESRs with no evidence of inflammation. Blood samples from the control group were handled and collected identically to the RIF group.

### VEGF-A and VEGF-R1 quantification:

The serum levels of both VEGF-A and VEGF-R1 were measured by ELISA, using commercially available kits (VEGF-A was detected by a Platinum ELISA kit; BMS277/2TEN, affymetrix eBioscience, and VEGF-R1 by a duoset ELISA: DY321B, R&D Systems). Their levels were determined by use of a standard curve and interpolation of concentrations on statistical software (GraphPad). The VEGF-R1 assay detected both cell surface VEGF receptor 1 as well as the circulating level of s-FLT-1.

### NK Cell Subtype Quantification:

This was undertaken according to our previously published methods (33). The method utilized whole blood analysis with red cell lysis using Easylyse (BD Biosciences) and with anti-CD56 PE, anti-CD16 FITC, anti-CD3 PE Cy5 and anti-CD69 APC (all supplied by BD Pharmingen). Flow cytometric analysis was undertaken on a Facscalibur (BD Biosciences), and using Cellquest Pro software. A minimum number of 10,000 cells were counted.

### NK Cell Cytotoxicity Assay:

This was performed according to standard methods in the St Helier Immunology Laboratory in Carshalton, Surrey. This protocol was based on the methods stated by Gilman-Sachs et al. (34). Briefly, PBMCs are isolated from whole blood, resuspended in culture medium and incubated for one hour in a 37°*C* and 5% CO_2_ incubator. The cells are then washed in pre-warmed culture medium, and the viable cell count is adjusted to a concentration of 5×10^6^/*ml*. Meanwhile, K562 cells are adjusted to a concentration of 2×10^5^/*ml* and subsequently working concentrations of Propidium Iodide are prepared. Different combinations of culture medium, PBMCs, K562 cells and Propidium Idioide were then arranged to achieve Effector: Target Cell Ratios of 50:1, 25:1 and 12:1. Following a 2 *hr* incubation in the 37°*C* 5% CO_2_ incubator, each tube was then analyzed flow cytometrically.

### Statistical Analysis:

Data was analyzed using GraphPad with Mann Whitney U test for unpaired non-parametric data and Spearman’s rank correlation for detecting any association between the VEGF-A/VEGF-R1 and age, NK cell activation, NK cell phenotype and also NK cell cytotoxicity results.

## Results

### VEGF-A Levels:

**Figure 2.**
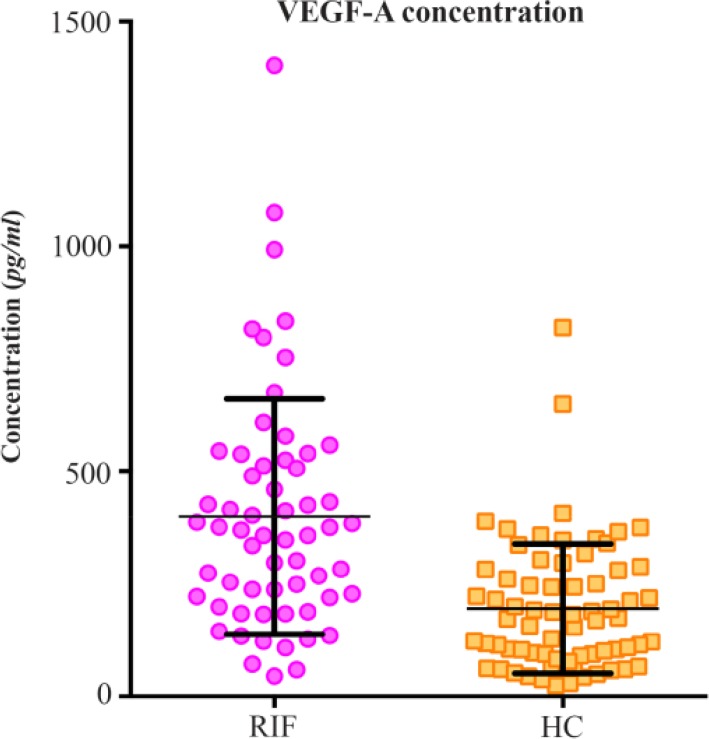
Serum VEGF-A Concentration in the Recurrent IVF failure and Healthy Control Groups

The serum VEGF-A, measured in 62 women with RIF was significantly raised compared to the 72 healthy control (HC) women [median 362.9 *pg/ml* (IQR 219.9–521.1 *pg/ml*), *vs*. 171.6 *pg/ml* (IQR 91.99–280.8 *pg/ml*), p<0.0001] (Figure 2). There was no relationship evident between age and VEGF-A levels in both groups (Spearman correlation r values were 0.1134 and 0.003348 with corresponding two-tailed p-values of 0.3881 and 0.9782 in the RIF and the HC groups, respectively).

### VEGF-R1 Levels:

**Figure 3.**
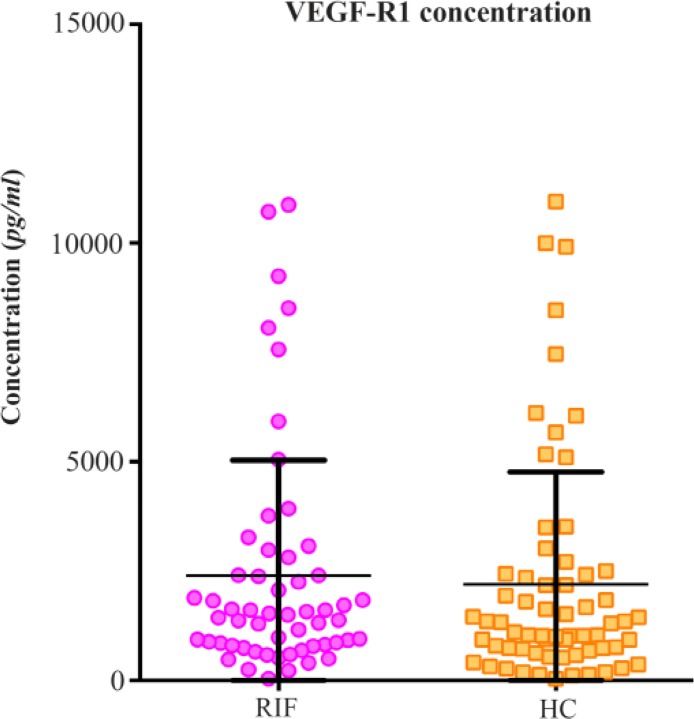
Soluble VEGF-R1 Concentration in the Recurrent IVF failure and Healthy Control Groups

The serum soluble VEGF-R1 levels in the RIF group were similar to that in the HC group depicted in figure 3 [median 1499 *pg/ml* (IQR 812.1–2608 *pg/ml*) *vs*. 1202 *pg/ml* (IQR 691.6–2429 *pg/ml*)]. As such there was no statistical significance seen with Mann-Whitney testing, with a p value of 0.4082.

### NK Cell Phenotyping and Activation:

The median total NK count was 9.25% (IQR 7–12.95%), the median NK CD69 count was 0.555×10^6^/*L* (IQR 0.4–0.7925×10^6^/*L*), and finally the median NK CD69^bright^ levels were 0.14×10^6^/*L* (IQR 0.07–0.3225×10^6^/*L*). There was no correlation between these results and the corresponding VEGF-A and VEGF-R1 levels. Spearman correlation testing for VEGF-A levels compared to total NK counts, total NK CD69 counts and total NK CD69^bright^ counts were as follows; r values with corresponding 2-tailed p-values of −0.1029 (p=0.4340), 0.1409 (p=0.2830) and 0.1803 (p=0.1680), respectively.

The Spearman correlation values for the VEGF-R1 levels with total NK counts, total NK CD69 and total NK CD69^bright^ were as follows; r=0.1754 (p=0.1918), r=− 0.1605 (p=0.2329) and r=0.1004 (p=0.4576), respectively.

### NK Cell Cytotoxicity:

The median killing was 27.5% (IQR 22–38%) at 50:1, 19.5% (IQR 16–29%) at 25:1 and 12% (IQR 8.75–18%) at 12.5:1. There was no correlation between the NK cytotoxicity values at each effector: target ratio and the corresponding VEGF-A and VEGF-R1 levels. For VEGF-A and NK, cytotoxicity killing ratios were 50:1, 25:1 and 12.5:1, r=0.04334 (p= 0.7423), r=− 0.02273 (p=0.8631) and r=− 0.02247 (p=0.8647), respectively. The same values for VEGF-R1 were r=0.1925 (p=0.1515) at 50:1, r= 0.1794 (p=0.1817) at 25:1, and r=0.1255 (p= 0.3525) at 12.5:1.

## Discussion

Successful pregnancy despite multiple embryo transfer remains stubbornly low at roughly one third of all IVF cycles. Unsuccessful IVF in some women may be attributed to a failure of implantation and possibly very early miscarriage. This may unfortunately be repeated in several consecutive cycles. There are several reports of changes in decidual vascularization in women with miscarriage. Compared to matched controls, the latter have shown fewer vessels of larger circumference and correlation with the expression of certain angiogenic factors and proteases (35).

The endometrial expression of pro-inflammatory cytokines such as IL-1β and TNF-α and also PGE2 have been found to be up-regulated with a reduction in the anti-inflammatory IL-10 in women with idiopathic RM. Furthermore, VEGF, endothelial nitric oxide synthase, nitric oxide and adrenomedullin were found to be down-regulated and correlated with impaired subendometrial blood flow (36). Thus, vasoactive factors are considered of major importance in early placentation and the delivery of oxygen and nutrients to the developing embryo/fetus.

Overall, there is general agreement that VEGF is particularly important in new vessel formation and placentation, which if inadequate, predisposes to miscarriage and other obstetric complications. This is supported by the reduced immunochemical expression of VEGF and its receptors in placental and decidual tissue of women with spontaneous incomplete abortion and missed abortion (37). This was also noted by Lash et al. (38), who in addition observed increased immunochemical staining for VEGF-R1 and VEGF-R2 in the vascular smooth muscle cells of cycling endometrium of women with a history of RM. Corroboration of these findings is also provided by much of the VEGF genetic data in women with RM which show an increased prevalence of those SNPs associated with reduced VEGF. For at least the −460T/C, 398G/A and − 583T/C genotypes there was a significant correlation with serum VEGF levels which overall was reduced in women with recurrent spontaneous miscarriage (39).

Increased plasma VEGF but normal levels of both VEGF-R1 and sFLT-1 were observed in women with RIF in whom only a very small proportion had RM. Unfortunately, the proportion of the detected VEGF and VEGF-R1 was impossible to be determined that was present as a free protein and was present as VEGF/VEGF-R1 complex. Our efforts to detect the presence of these complexes by coating ELISA plates with antibodies capturing VEGF or VEGF-R1 and then detecting with Alkaline phosphatase conjugated anti-VEGF-R1 or VEGF antibodies produced very low optical density readings. While this may be due to the absence of these complexes, it is of course possible that the assays were ineffective at either the capture or detection phases and there were no positive controls to confirm the working of the assays. Nonetheless, the raised plasma VEGF in our patients with RIF remains unexplained given that RM is generally associated with lowered levels of circulating VEGF and RIF can in some ways be seen as very early miscarriage. However, raised VEGF was also noted by Amirchaghmaghi et al. (40) in the serum of women with RM collected between day 19 and 26 of the menstrual cycle. Similarly, in women with threatened abortion serum VEGF and soluble VEGF-R1 (s-FLT) was significantly elevated relative to healthy control pregnancies of similar gestational age (25). Earlier, Pang et al. (26) found both VEGF and sFLT to be significantly raised in women in early pregnancy compared to those who were not pregnant. Interestingly, the levels of both factors were significantly higher in women who subsequently miscarried. This is in contrast to the reduced levels of serum sFLT reported by Muttukrishna et al. (41) in women with threatened miscarriage compared to pregnant women with a subsequent good outcome. There has also been an inexplicably raised serum VEGF found in women with ectopic pregnancy compared to those with a normal pregnancy (42). Thus, the data surrounding assessment of circulating VEGF and sFLT levels appears to be different to that suggested by genetic studies and from decidual immunochemical expression. It is speculated whether raised levels of circulating VEGF in the presence of normal levels of sFLT in women with RIF may represent compensation for a deficiency of other factors that are necessary for placentation. The latter may include preimplantation factor (43), which interestingly has been shown to reduce NK cell cytotoxicity and NK CD69 expression. Alternatively, angiogenesis is not as important in RIF as it is in RM and maternal immune-mediated embryotoxicity, possibly via activated NK cells (44) or raised Th1 type immunity (45) being a more important factor. Finally, it is of course possible that the raised VEGF-A in the RIF/RM patients may represent an attempt to overcome impaired VEGF-R1 activity that is required for angiogenesis.

It was previously found that women with RIF have significantly increased activated NK cells (18). NK cells have also been shown to express and release VEGF. Thus in this study, the relationship between these important variables was investigated in women with RIF to see if the raised plasma VEGF may perhaps be linked to several NK cell variables. However, no correlation was found between circulating levels of VEGF and the absolute numbers of NK cells, activated NK cells or NK cytotoxicity. Thus, our data suggests that these variables may be acting independently in influencing the success of IVF and that the plasma VEGF may perhaps be produced by cells other than peripheral blood NK cells, such as macrophages and endothelial cells.

While the level of VEGF has been reported as raised in endometriosis, this is unlikely to explain our observation of raised plasma VEGF in women with RIF, as only 4% had endometriosis (46). It is speculated whether the raised VEGF represents a maternal response to support an embryo that is threatened by presently unidentified anti-angiogenic factors. Alternatively, significantly elevated levels of VEGF may disrupt normal angiogenesis through an overstimulation of blood vessels leading to disturbed vascular architecture (47).

In conclusion, a significantly raised level of plasma VEGF was found in women with RIF but without correlation with several NK cell variables. It is presently unclear why plasma VEGF levels are raised in RIF when there is much evidence suggesting impaired angiogenesis in this and in RM.

## References

[1] Tan BK Vandekerckhove P Kennedy R Keay SD Investigation and current management of recurrent IVF treatment failure in the UK. BJOG. 2005;112 (6):773–80. 15924536 10.1111/j.1471-0528.2005.00523.x

[2] Polanski LT Barbosa MA Martins WP Baumgarten MN Campbell B Brosens J Interventions to improve reproductive outcomes in women with elevated natural killer cells undergoing assisted reproduction techniques: a systematic review of literature. Hum Reprod. 2014;29(1):65–75. 24256994 10.1093/humrep/det414

[3] Llurba E Crispi F Verlohren S Update on the pathophysiological implications and clinical role of angiogenic factors in pregnancy. Fetal Diagn Ther. 2015;37(2):81–92. 25659427 10.1159/000368605

[4] Muttukrishna S Suri S Groome N Jauniaux E Relationships between TGFbeta proteins and oxygen concentrations inside the first trimester human gestational sac. PLoS One. 2008;3(6):e2302. 18523681 10.1371/journal.pone.0002302 PMC2409140

[5] Weimar CH Kavelaars A Brosens JJ Gellersen B de Vreeden-Elbertse JM Heijnen CJ Endometrial stromal cells of women with recurrent miscarriage fail to discriminate between high- and low-quality human embryos. PLoS One. 2012;7(7): e41424. 22848492 10.1371/journal.pone.0041424 PMC3405140

[6] Duffy AM Bouchier-Hayes DJ Harmey JH Vascular endothelial growth factor (VEGF) and its role in non-endothelial cells: Autocrine signalling by VEGF. In: Madame Curie Bioscience Database [Internet]. Austin (TX): Landes Bioscience; 2000–2013 Available from: https://www.ncbi.nlm.nih.gov/books/NBK6482/

[7] Li XF Charnock-Jones DS Zhang E Hiby S Malik S Day K Angiogenic growth factor messenger ribonucleic acids in uterine natural killer cells. J Clin Endocrinol Metab. 2001;86(4):1823–34. 11297624 10.1210/jcem.86.4.7418

[8] Charnock-Jones DS Kaufmann P Mayhew TM Aspects of human fetoplacental vasculogenesis and angiogenesis. I. Molecular regulation. Placenta. 2004; 25(2–3):103–13. 14972443 10.1016/j.placenta.2003.10.004

[9] Taylor CM Stevens H Anthony FW Wheeler T Influence of hypoxia on vascular endothelial growth factor and chorionic gonadotrophin production in the trophoblast-derived cell lines: JEG, JAr and BeWo. Placenta. 1997;18(5–6):451–8. 9250709 10.1016/s0143-4004(97)80047-1

[10] Waltenberger J Claesson-Welsh L Siegbahn A Shibuya M Heldin CH Different signal transduction properties of KDR and Flt1, two receptors for vascular endothelial growth factor. J Biol Chem. 1994;269(43):26988–95. 7929439

[11] Gerber HP Condorelli F Park J Ferrara N Differential transcriptional regulation of the two vascular endothelial growth factor receptor genes. Flt-1, but not Flk-1/KDR, is up-regulated by hypoxia. J Biol Chem. 1997;272(38):23659–67. 9295307 10.1074/jbc.272.38.23659

[12] Ratsep MT Felker AM Kay VR Tolusso L Hofmann AP Croy BA Uterine natural killer cells: supervisors of vasculature construction in early decidua basalis. Reproduction. 2015;149(2):R91–102. 25342175 10.1530/REP-14-0271

[13] Robson A Harris LK Innes BA Lash GE Aljunaidy MM Aplin JD Uterine natural killer cells initiate spiral artery remodeling in human pregnancy. FASEB J. 2012;26(12):4876–85. 22919072 10.1096/fj.12-210310

[14] Fu B Li X Sun R Tong X Ling B Tian Z Natural killer cells promote immune tolerance by regulating inflammatory TH17 cells at the human maternal-fetal interface. Proc Natl Acad Sci USA. 2013;110(3):E231–40. 23271808 10.1073/pnas.1206322110 PMC3549088

[15] Gao Y Wang PL Increased CD56(+) NK cells and enhanced Th1 responses in human unexplained recurrent spontaneous abortion. Genet Mol Res. 2015; 14(4):18103–9. 26782457 10.4238/2015.December.22.36

[16] Karami N Boroujerdnia MG Nikbakht R Khodadadi A Enhancement of peripheral blood CD56 (dim) cell and NK cell cytotoxicity in women with recurrent spontaneous abortion or in vitro fertilization failure. J Reprod Immunol. 2012;95(1–2):87–92. 22854126 10.1016/j.jri.2012.06.005

[17] Ghafourian M Karami N Khodadadi A Nikbakht R Increase of CD69, CD161 and CD94 on NK cells in women with recurrent spontaneous abortion and in vitro fertilization failure. Iran J Immunol. 2014;11(2):84–96. 24975965 10.22034/iji.2014.16769

[18] Thum MY Bhaskaran S Abdalla HI Ford B Sumar N Shehata H An increase in the absolute count of CD56dimCD16+CD69+ NK cells in the peripheral blood is associated with a poorer IVF treatment and pregnancy outcome. Hum Reprod. 2004;19(10):2395–400. 15319390 10.1093/humrep/deh378

[19] Papamitsou T Toskas A Papadopoulou K Sioga A Lakis S Chatzistamatiou M Immunohistochemical study of immunological markers: HLAG, CD16, CD25, CD56 and CD68 in placenta tissues in recurrent pregnancy loss. Histol Histopathol. 2014;29(8):1047–55. 24557735 10.14670/HH-29.1047

[20] Wang S Li YP Ding B Zhao YR Chen ZJ Xu CY Recurrent miscarriage is associated with a decline of decidual natural killer cells expressing killer cell immunoglobulin-like receptors specific for human leukocyte antigen C. J Obstet Gynaecol Res. 2014;40(5):1288–95. 24689450 10.1111/jog.12329

[21] Almasry SM Elmansy RA Elfayomy AK Algaidi SA Ultrastructure alteration of decidual natural killer cells in women with unexplained recurrent miscarriage: a possible association with impaired decidual vascular remodelling. J Mol Histol. 2015; 46(1):67–78. 25355193 10.1007/s10735-014-9598-8

[22] Jakovljevic A Bogavac M Lozanov-Crvenkovic Z Milosević-Tosic M Nikolic A Mitic G Early pregnancy angiogenic proteins levels and pregnancy related hypertensive disorders. J Matern Fetal Neonatal Med. 2017;30(5):534–9. 27109751 10.1080/14767058.2016.1177814

[23] Andraweera PH Dekker GA Roberts CT The vascular endothelial growth factor family in adverse pregnancy outcomes. Hum Reprod Update. 2012; 18(4):436–57. 22495259 10.1093/humupd/dms011

[24] Maynard SE Min JY Merchan J Lim KH Li J Mondal S Excess placental soluble fms-like tyrosine kinase 1 (sFlt1) may contribute to endothelial dysfunction, hypertension, and proteinuria in preeclampsia. J Clin Invest. 2003;111(5):649–58. 12618519 10.1172/JCI17189 PMC151901

[25] Keskin U Ulubay M Dede M Ozgurtas T Koçyigit YK Aydin FN The relationship between the VEGF/sVEGFR-1 ratio and threatened abortion. Arch Gynecol Obstet. 2015;291(3):557–61. 25200689 10.1007/s00404-014-3452-9

[26] Pang L Wei Z Li O Huang R Qin J Chen H An increase in vascular endothelial growth factor (VEGF) and VEGF soluble receptor-1 (sFlt-1) are associated with early recurrent spontaneous abortion. PLoS One. 2013;8(9):e75759. 24098721 10.1371/journal.pone.0075759 PMC3787054

[27] Xu X Du C Li H Du J Yan X Peng L Association of VEGF genetic polymorphisms with recurrent spontaneous abortion risk: a systematic review and meta-analysis. PLoS One. 2015;10(4): e0123696. 25894555 10.1371/journal.pone.0123696 PMC4404341

[28] Yalcintepe SA Silan F Hacivelioglu SO Uludag A Cosar E Ozdemir O Fetal Vegf Genotype is More Important for Abortion Risk than Mother Genotype. Int J Mol Cell Med. 2014;3(2):88–94. 25035858 PMC4082810

[29] Chiossone L Vacca P Orecchia P Croxatto D Damonte P Astigiano S In vivo generation of decidual natural killer cells from resident hematopoietic progenitors. Haematologica. 2014;99(3): 448–57. 24179150 10.3324/haematol.2013.091421 PMC3943307

[30] Lima PD Tu MM Rahim MM Peng AR Croy BA Makrigiannis AP Ly49 receptors activate angiogenic mouse DBA^+^ uterine natural killer cells. Cell Mol Immunol. 2014;11(5):467–76. 24954223 10.1038/cmi.2014.44 PMC4197209

[31] Zhang J Dunk CE Lye SJ Sphingosine signalling regulates decidual NK cell angiogenic phenotype and trophoblast migration. Hum Reprod. 2013;28 (11):3026–37. 24001716 10.1093/humrep/det339

[32] Chiba H Fukui A Fuchinoue K Funamizu A Tanaka K Mizunuma H Expression of Natural Cytotoxicity Receptors on and Intracellular Cytokine Production by NK Cells in Women with Gestational Diabetes Mellitus. Am J Reprod Immunol. 2016;75(5):529–38. 26813019 10.1111/aji.12491

[33] Mosimann B Wagner M Shehata H Poon LC Ford B Nicolaides KH Natural killer cells and their activation status in normal pregnancy. Int J Reprod Med. 2013;2013:906813. 25763390 10.1155/2013/906813 PMC4334074

[34] Gilman-Sachs A DuChateau BK Aslakson CJ Wohlgemuth GP Kwak JY Beer AE Natural killer (NK) cell subsets and NK cell cytotoxicity in women with histories of recurrent spontaneous abortions. Am J Reprod Immunol. 1999;41(1):99–105. 10097793 10.1111/j.1600-0897.1999.tb00081.x

[35] Plaisier M Dennert I Rost E Koolwijk P van Hinsbergh VW Helmerhorst FM Decidual vascularization and the expression of angiogenic growth factors and proteases in first trimester spontaneous abortions. Hum Reprod. 2009;24(1):185–97. 18854409 10.1093/humrep/den296

[36] Banerjee P Ghosh S Dutta M Subramani E Khalpada J Roychoudhury S Identification of key contributory factors responsible for vascular dysfunction in idiopathic recurrent spontaneous miscarriage. PLoS One. 2013;8(11):e80940. 24260517 10.1371/journal.pone.0080940 PMC3829935

[37] Col-Madendag I Madendag Y Altinkaya SÖ Bayramoglu H Danisman N The role of VEGF and its receptors in the etiology of early pregnancy loss. Gynecol Endocrinol. 2014;30(2):153–6. 24303885 10.3109/09513590.2013.864272

[38] Lash GE Innes BA Drury JA Robson SC Quenby S Bulmer JN Localization of angiogenic growth factors and their receptors in the human endometrium throughout the menstrual cycle and in recurrent miscarriage. Hum Reprod. 2012;27(1):183–95. 22081249 10.1093/humrep/der376

[39] Almawi WY Saldanha FL Mahmood NA Al-Zaman I Sater MS Mustafa FE Relationship between VEGFA polymorphisms and serum VEGF protein levels and recurrent spontaneous miscarriage. Hum Reprod. 2013;28(10):2628–35. 23900206 10.1093/humrep/det308

[40] Amirchaghmaghi E Rezaei A Moini A Roghaei MA Hafezi M Aflatoonian R Gene expression analysis of VEGF and its receptors and assessment of its serum level in unexplained recurrent spontaneous abortion. Cell J. 2015;16(4):538–45. 25685744 10.22074/cellj.2015.498 PMC4297492

[41] Muttukrishna S Swer M Suri S Jamil A Calleja-Agius J Gangooly S Soluble Flt-1 and PlGF: new markers of early pregnancy loss? PLoS One. 2011;6(3):e18041. 21448460 10.1371/journal.pone.0018041 PMC3063178

[42] Fernandes da Silva MO Elito J Jr Daher S Camano L Fernandes Moron A Association of serum levels of vascular endothelial growth factor and early ectopic pregnancy. Clin Exp Obstet Gynecol. 2013;40(4):489–91. 24597240

[43] Roussev RG Dons’koi BV Stamatkin C Ramu S Chernyshov VP Coulam CB Preimplantation factor inhibits circulating natural killer cell cytotoxicity and reduces CD69 expression: implications for recurrent pregnancy loss therapy. Reprod Biomed Online. 2013;26(1):79–87. 23186554 10.1016/j.rbmo.2012.09.017

[44] Miko E Manfai Z Meggyes M Barakonyi A Wilhelm F Varnagy A Possible role of natural killer and natural killer T-like cells in implantation failure after IVF. Reprod Biomed Online. 2010;21 (6):750–6. 21051289 10.1016/j.rbmo.2010.07.012

[45] Kalu E Bhaskaran S Thum MY Vishwanatha R Croucher C Sherriff E Serial estimation of Th1:th2 cytokines profile in women undergoing in-vitro fertilization-embryo transfer. Am J Reprod Immunol. 2008;59(3):206–11. 18275514 10.1111/j.1600-0897.2007.00565.x

[46] Vodolazkaia A Yesilyurt BT Kyama CM Bokor A Schols D Huskens D Vascular endothelial growth factor pathway in endometriosis: genetic variants and plasma biomarkers. Fertil Steril. 2016;105(4):988–96. 26773192 10.1016/j.fertnstert.2015.12.016

[47] Sergent F Hoffmann P Brouillet S Garnier V Salomon A Murthi P Sustained Endocrine Gland-Derived Vascular Endothelial Growth Factor Levels Beyond the First Trimester of Pregnancy Display Phenotypic and Functional Changes Associated With the Pathogenesis of Pregnancy-Induced Hypertension. Hypertension. 2016;68(1): 148–56. 27141059 10.1161/HYPERTENSIONAHA.116.07442

